# A Symmetric Region of the HIV-1 Integrase Dimerization Interface Is Essential for Viral Replication

**DOI:** 10.1371/journal.pone.0045177

**Published:** 2012-09-18

**Authors:** Erik Serrao, Wannes Thys, Jonas Demeulemeester, Laith Q. Al-Mawsawi, Frauke Christ, Zeger Debyser, Nouri Neamati

**Affiliations:** 1 Department of Pharmacology and Pharmaceutical Sciences, School of Pharmacy, University of Southern California, Los Angeles, California, United States of America; 2 Laboratory for Molecular Virology and Gene Therapy, Division of Molecular Medicine, Katholieke Universiteit Leuven, Flanders, Belgium; Centro de Biología Molecular Severo Ochoa (CSIC-UAM), Spain

## Abstract

HIV-1 integrase (IN) is an important target for contemporary antiretroviral drug design research. Historically, efforts at inactivating the enzyme have focused upon blocking its active site. However, it has become apparent that new classes of allosteric inhibitors will be necessary to advance the antiretroviral field in light of the emergence of viral strains resistant to contemporary clinically used IN drugs. In this study we have characterized the importance of a close network of IN residues, distant from the active site, as important for the obligatory multimerization of the enzyme and viral replication as a whole. Specifically, we have determined that the configuration of six residues within a highly symmetrical region at the IN dimerization interface, composed of a four-tiered aromatic interaction flanked by two salt bridges, significantly contributes to proper HIV-1 replication. Additionally, we have utilized a quantitative luminescence assay to examine IN oligomerization and have determined that there is a very low tolerance for amino acid substitutions along this region. Even conservative residue substitutions negatively impacted IN multimerization, resulting in an inactive viral enzyme and a non-replicative virus. We have shown that there is a very low tolerance for amino acid variation at the symmetrical dimeric interface region characterized in this study, and therefore drugs designed to target the amino acid network detailed here could be expected to yield a significantly reduced number of drug-resistant escape mutations compared to contemporary clinically-evaluated antiretrovirals.

## Introduction

HIV-1 integrase (IN) is an enzyme essential for viral replication. After more than a decade of intensive research, the first IN drug - raltegravir – was approved by the FDA in October of 2007. This advance has been a major achievement, but like other HIV-1 antiretroviral drugs targeting reverse transcriptase (RT) and protease, considerable resistance has already emerged following clinical use [1], [2], [3], [4]. IN is critical for the viral life cycle, as it acts to integrate the viral DNA into the host cell chromosomal material. The resulting integrated provirus is invulnerable to current antiretrovirals, and upon upregulation of certain cellular transcription factors, the provirus can be replicated by host cell machinery to generate progeny virus [5], [6], [7]. The provirus can also remain dormant for years in memory T-cells, greatly contributing to the difficulty in eradicating viral infection. In fact, it has been shown that very low levels of HIV-1 transcription can persist in peripheral blood mononuclear cells in patients receiving antiretroviral therapy, exacerbating the problem of emerging drug-resistant viral strains [8], [9], [10], [11].

IN exists as a monomer, dimer, and higher oligomers in solution, and multimerization is essential for its catalytic activity [12], [13], [14]. The amino acid network at the IN dimeric interface is extensive, and it is stabilized by both hydrophobic and electrostatic interactions between four α -helices (α1, α3, α5, and α6) from each monomer, and an additional subunit interface interaction donated by a β-strand from each monomer [5], [15], [16]. It contains three distinct domains: an N-terminal domain (residues 1–50) that binds zinc, a catalytic core domain (residues 50–212) that contains the active site DD(35)E motif and many residues essential for dimerization, and a C-terminal domain (residues 213–288) that possesses nonspecific DNA affinity and is important for IN tetramerization [17], [18], [19]. After viral entry into the host cell, IN associates with RT, the viral RNA genome, and multiple other viral and cellular proteins in a large nucleoprotein complex termed the reverse transcription complex [20], [21]. After reverse transcription is completed, IN cleaves a dinucleotide from the 3′ end of the newly-formed viral DNA at a conserved CA sequence to yield a reactive hydroxyl moiety via a cytosolic reaction termed 3′-processing [22]. IN, in complex with the processed viral DNA and viral and host proteins, forms another large nucleoprotein assembly termed the preintegration complex (PIC) [23]. The PIC enters the nucleus through the nuclear pore, and IN then adheres to the host cell chromatin with the assistance of the cellular cofactor LEDGF/p75 [24], [25]. Once tethered to the host cell chromatin, IN utilizes the free 3′-hydroxyl group of the viral DNA in a nucleophilic attack upon the host genome largely within transcriptionally active regions [26] to stably integrate the proviral DNA, a reaction termed strand transfer [26], [27], [28], [29]. IN uses the same active site to catalyze both the 3′-processing and strand transfer reactions by coordinating two Mg^2+^ ions with three critical acidic residues (Asp^64^, Asp^116^, and Glu^152^) within the active site (DD(35)E motif) [30], [31]. Rational drug design efforts have thus far been mainly directed toward developing compounds that bind to the Mg^2+^-coordinating active site, but it has become apparent that new classes of allosteric inhibitors that disrupt IN-cofactor interactions [32], [33] or IN multimerization [34] will be necessary to advance the antiretroviral field in light of the emergence of viral strains resistant to contemporary clinically used IN drugs.

Although there have been scant structural studies focusing directly on the IN dimeric interface [35], [36], [37], and a handful of studies aimed at abrogating or modulating multimerization using peptidic or small-molecule compounds [34], [38], [39], [40], [41], [42], [43], [44], [45], relatively little is currently known about the inhibition of IN catalysis through blocking its oligomerization. Traditional small molecule drug design programs aimed at disrupting protein-protein interactions have been hindered by the belief that most drug-like small molecules do not provide a high enough binding energy to bind and disrupt large interfacial protein hotspots. However, great advances have been made in both antiretroviral and cancer drug design targeting protein-protein hotspots [32], [46]. Previously, our group identified an allosteric site of inhibition at the dimeric interface of IN [47]. Specifically, we found that a photoaffinity-labeled coumarin compound disrupted proper IN multimerization and, therefore, delivered inhibition of IN catalytic activities via an allosteric inhibitory mechanism. In a follow up study to further characterize the IN interfacial dimeric region, we identified a highly symmetric amino acid network composed of a four-tiered aromatic interaction flanked by two charged centers, with contributions from both monomers of the IN dimer [48]. In the current study, we aimed to further analyze the importance of each amino acid involved in this IN hotspot for oligomerization. Through site-directed mutagenesis, we determined that all but one substitution made at the dimeric interface abolished *in vitro* enzymatic activity of IN. Furthermore, we used a quantitative AlphaScreen®-based assay [49] to study the contribution of substituted residues to IN multimerization – a platform that has already proved potentially viable for future identification of IN multimerization-disrupting inhibitors [45]. Using this assay we validated our enzymatic activity results by showing that only the active IN mutant was able to functionally oligomerize. Many of the IN mutants detailed in this study proved difficult to purify and concentrate through traditional biochemical techniques, and thus the present study utilized IN inclusion bodies to analyze the strand transfer activity of the mutant proteins in the presence of Mn^2+^. Furthermore, we show that IN mutants exhibiting defective oligomerization can exhibit some degree of strand transfer *in vitro*. We went further to generate IN mutants in the NL4.3 viral clone and showed that, although exogenous RT activity mostly resembled WT levels, dimeric interface mutant viruses were non-infectious. We conclude that the structural conformation of the amino acid network detailed in this study is important for HIV-1 replication, and even conservative mutations at this region are not tolerable.

## Results

### IN Mutants with a Disrupted Four-tiered Dimeric Interface Interaction are Inactive in vitro

**Figure 1:**
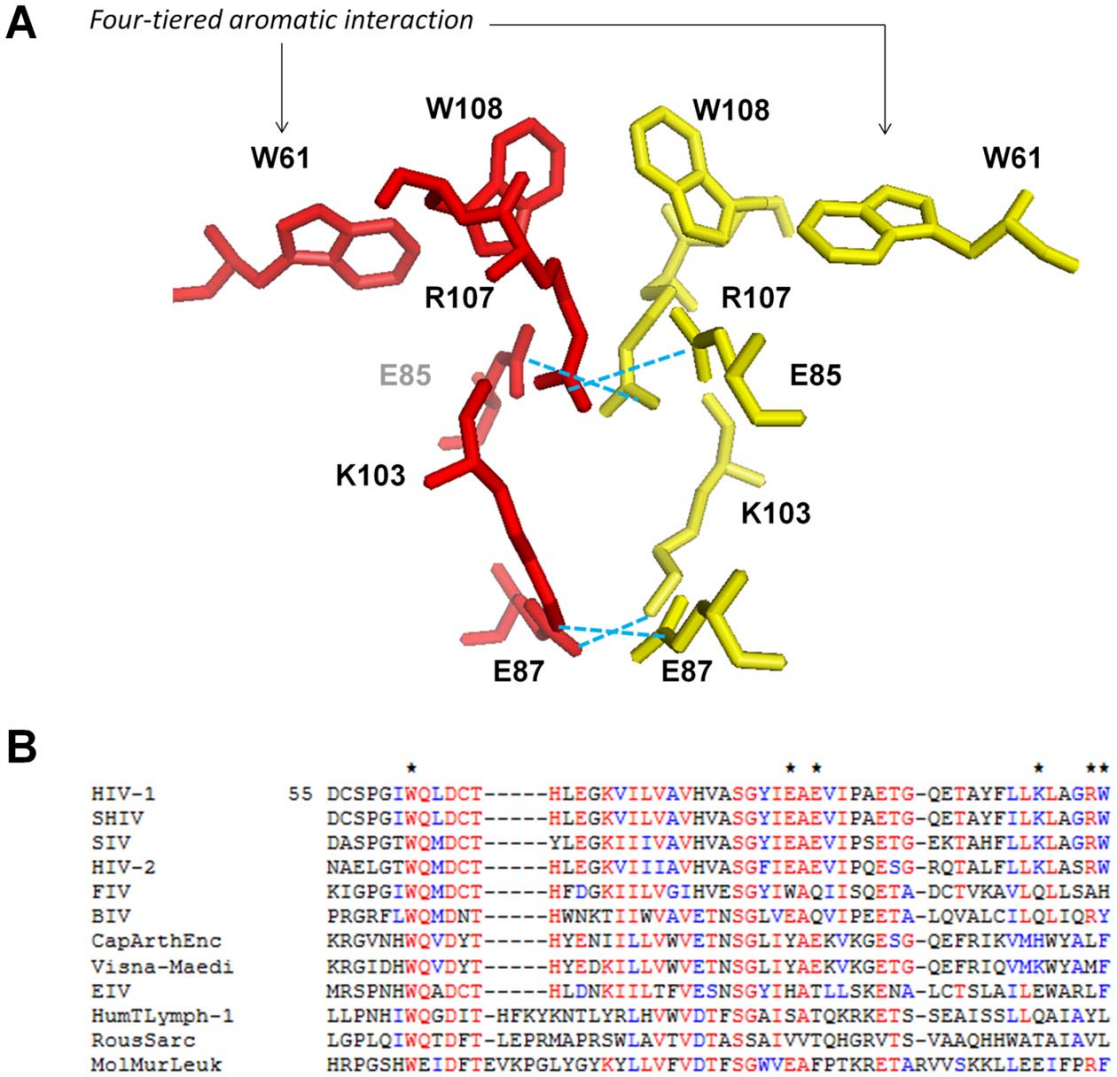
A symmetric region of the HIV-1 dimerization interface is conserved across other lentiviruses. (A) PyMOL representation of the highly symmetric region at the HIV-1 IN dimeric interface. A four-tiered aromatic interaction between W61 and W108 from each IN monomer is flanked by two salt bridges composed of E85 and R107, and E87 and K103 from each monomer. The four-tiered aromatic interaction donates at least −10 kcal/mol of stabilization energy to the interface. (B) Sequence alignment of relevant lentiviral IN residues, beginning at IN residue 55. Red text denotes highly conserved residues, while blue signifies moderately conserved. W61, E85, E87, K103, R107, and W108 are all completely conserved throughout HIV-1, SHIV, SIV, and HIV-1 viruses. Aromaticity is heavily conserved across most lentiviruses for positions 61 and 108.

Depicted in Figure 1A is a PyMOL (W.L. DeLano, www.pymol.org) representation of the symmetrical amino acid network along the IN dimeric interface, which consists of a four-tiered aromatic interaction flanked by two charged centers. Residues W61 and W108 of each IN monomer form T-shaped edge-to-face stacking interactions, and we have previously determined that the average distance between each tryptophan is highly balanced: 4.54 Å for W61 (monomer A)-W108 (monomer A), 4.42 Å for W108 (monomer A)-W108 (monomer B), and 4.54 Å for W108 (monomer B)-W61 (monomer B). We calculated the amount of stabilization energy that this four-tiered interaction donates to the dimeric interface to be at least −10 kcal/mol [48] if each amino-acid interaction is considered to provide an additive effect, however the true stabilizing energy contribution is likely greater as extensive p-orbital pi-interactions are known to be synergistic. Also depicted are residues E87 and K103, along with E85 and R107, which form two salt-bridge linkages between the two IN monomers. Sequence alignment analysis (Figure 1B) has revealed a high degree of conservation among lentiviral integrases for all of the residues under study.

To probe the importance of these residues in the stability of the HIV-1 IN dimeric interface, we first constructed conservative and non-conservative substitutions and tested their catalytic activity in an *in vitro* enzymatic activity assay. Specifically, we synthesized W61F, W61G, E85F, E85G, E87F, E87G, K103E, K103G, R107E, R107G, W108F, and W108G IN mutant proteins. Unfortunately, most of the above IN mutations generated insoluble proteins with negligible yields after traditional nickel affinity chromatography. Though it has recently been shown that WT HIV-1 IN may be purified in the monomeric form [50], we postulated that the reduced yields of our mutant IN proteins could be due to some degree of misfolding or an overall reduction in protein solubility. Therefore, in order to gain some insight into the catalytic activity of the wild-type and mutant IN proteins simultaneously, we tested the protein activity within processed IN mutant lysate preparations. Specifically, after inducing IN expression and lysing the expression cells, we proceeded directly to homogenization and dialysis of isolated inclusion bodies from the wild-type and mutant IN protein lysates (see Methods).

**Figure 2:**
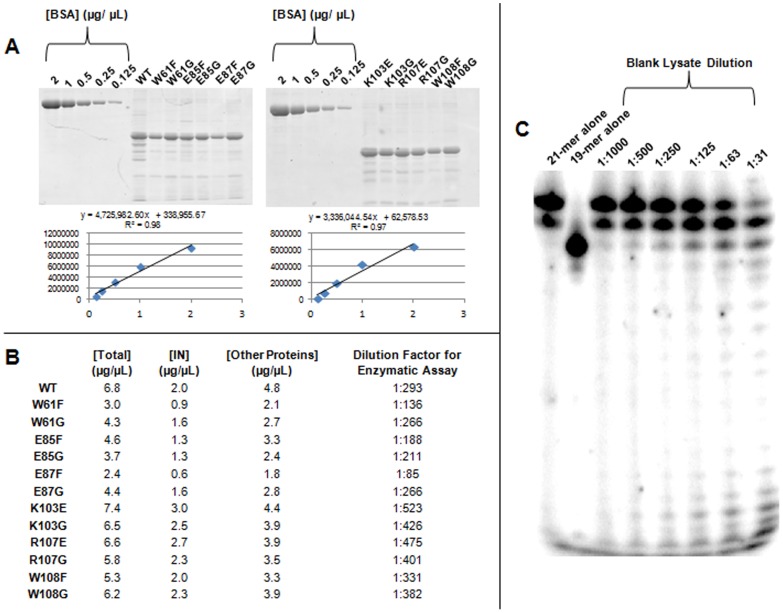
Cellular proteins in *E. coli* lysate confer a negligible level of non-specific DNA cutting. A) PAGE demonstrating the purity of each IN lysate preparation. Protein concentration standard curves were calculated from densitometric analysis of BSA titrations spanning five concentrations, from 0.125 µg/µL –2.0 µg/µL. Band intensity of IN within each total lysate was then quantified, and concentration of total IN was calculated using BSA standard curve line equation. B) Chart outlining total lysate concentration, IN concentration within lysate, and dilution factor necessary for 300 nM final IN concentration within enzymatic assay. Dilution factors necessary for each IN preparation ranged from 1∶85 (E87F) to 1∶475 (R107E). C) Effect of *E. coli* BL21(DE3)pLysS lysate on nonspecific nucleolytic degradation of radiolabeled 21-mer DNA. Radiolabeled 21-mer and 19-mer DNA oligonucleotide standards are shown in the first two lanes. *E. coli* cells not containing IN were incubated and IPTG induced under identical conditions as each IN lysate culture, and a lysate preparation was also manufactured in the same way. A titration of blank *E. coli* lysate concentrations spanning the range of dilutions made for the enzymatic assay was incubated with radiolabeled 21-mer, and resulting nonspecific DNA degradation was represented by a reduction of 21-mer band intensity and an increase of 20-mer, 19-mer, and other reduced size band intensities. Nonspecific DNA degradation by *E. coli* lysate proteins lost significance after a 1∶63 dilution. Since the lowest IN lysate dilution used in enzymatic assay was 1∶85, we are not concerned about confounding of results and conclusions due to nonspecific DNA degradation by non-IN (nuclease) proteins in our lysate preparations.

**Figure 3:**
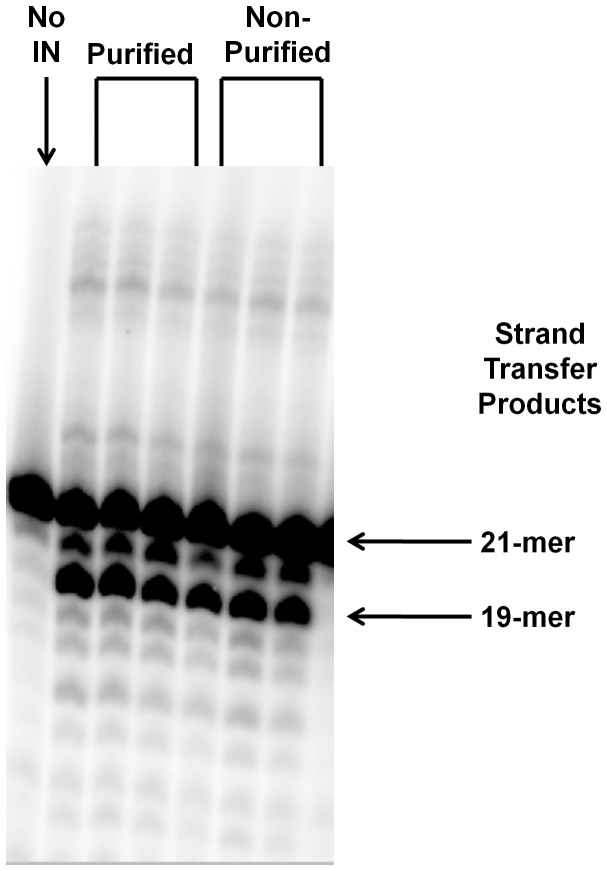
IN in inclusion body extracts is enzymatically active. The activity of IN in processed expression bacterial lysate is identical to that of purified IN, suggesting that full purification of IN is not a necessary step for *in vitro* analysis of function. Purified and processed INs were titrated from 200–600 nM. Both enzymes were highly active even at the lowest concentration tested. Identically processed expression cell lysate without IN (“No IN,” first lane) lacks nonspecific nuclease activity.

In an enzymatic assay that includes a γ-^32^P labeled 21-mer oligonucleotide substrate mimicking the HIV-1 U5 LTR DNA termini, we analyzed the activity of our WT and mutant IN proteins. Accordingly, we were compelled to discern whether potential nuclease activity from non-IN proteins in the *E. coli* lysate extracts would lead to nonspecific degradation of DNA products that would cloud our precise analysis of IN enzymatic activity. First, we computed the relative purity and concentration of each IN protein in our study through Coomassie PAGE followed by densitometric analysis of band intensity (Figure 2A&B). We observed similar relative abundance and purity of WT and all mutant IN proteins in this study. Though the IN extracts were not fully purified, the IN proteins in the extracts were optimally abundant due to IPTG induction during culture. This allowed for a high (at least 1∶85) extract dilution – and thus high dilution of non-IN proteins – to be utilized for subsequent enzymatic analysis of the enzymes. We then analyzed the effect of *E. coli* lysate-minus-IN on nonspecific degradation of radiolabeled 21-mer DNA oligonucleotide in the IN enzymatic assay. A non-IN-transformed *E. coli* culture was grown and processed identically to IN cultures, and lysate extract was titrated against 21-mer DNA (Figure 2C). We found that nonspecific degradation of the 21-mer oligonucleotide by non-IN proteins lost significance after a 1∶63 dilution. Our lowest IN dilution necessary for the enzymatic activity analysis that we present in Figure 3 below (final concentration of 300 nM) was 1∶85 (for the E87F mutant). It is noteworthy that even reactions including high dilutions of the blank lysate generated an increase in 20-mer DNA species, though 19-mer accumulation was insignificant.

Next, enzymatic activities of purified and inclusion body-isolated WT IN were compared to determine if this was a viable approach to analyze IN catalytic activity *in vitro*. As evident in Figure 3, the activity of IN is identical in both the purified and processed lysate forms, as both extracts were highly active even down to as low as 200 nM concentrations. Nonspecific 20-mer DNA cleavage product accumulation was similar for both purified and processed IN proteins. Alongside these samples we included a processed lysate-only sample (“No IN”) as an additional check against potential nonspecific DNA degradation resulting from possible nucleases in our IN preps. It is clear in the first lane of the Figure 3 gel that there is no significant nuclease contamination contributing to 19-mer or high molecular weight DNA banding (corresponding to the molecular weight of specific strand transfer banding) within our expression cell lysate. Nonspecific degradation leading to 20-mer accumulation was slight in this reaction.

**Figure 4:**
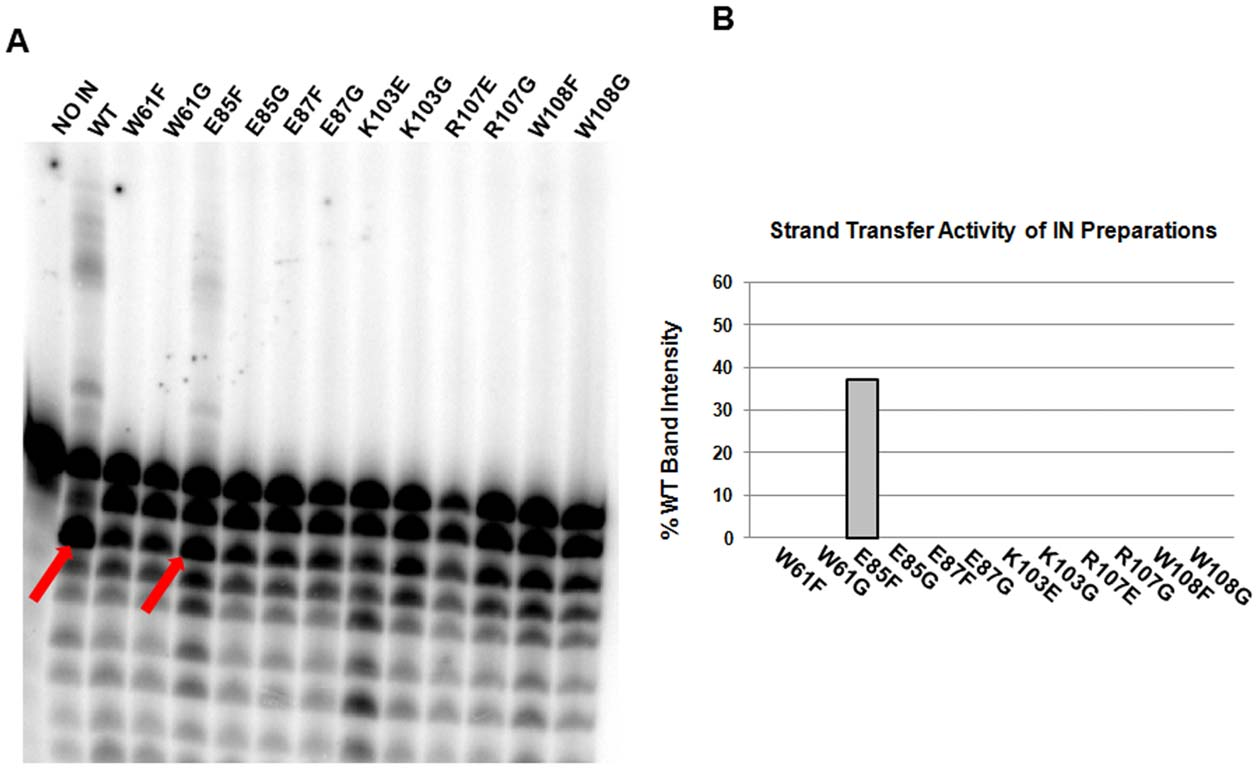
Dimeric interface mutations abrogate IN catalysis. (A) The strand transfer activity of partly-purified mutant IN proteins with 21-mer substrate. (B) E85F is the only mutant protein that exhibited strand transfer activity in the presence of radiolabeled 21-mer oligonucleotide substrate.

We next analyzed the *in vitro* strand transfer activity of each mutant IN and found that all substitutions within the tryptophan stacking interaction abolished strand transfer catalysis (Figure 4A). Interestingly, the E85F mutation of one charged center retained 37% of WT strand transfer activity (Figure 4B), but the non-conservative uncharged substitution E85G was totally inactive. Similarly, all substitutions made at E87, K103, and R107 resulted in negligible strand transfer activity. The data in Figure 4A indicate a significant nuclease presence in the IN extracts, and therefore 3′-processing activities of the IN mutants could not be quantitatively evaluated with high confidence. Addition of 50 µM ZnCl_2_, which has previously been shown to enhance IN multimerization and activity *in vitro* [51], [52], had no stimulatory effect on mutant activity (data not shown).

**Figure 5:**
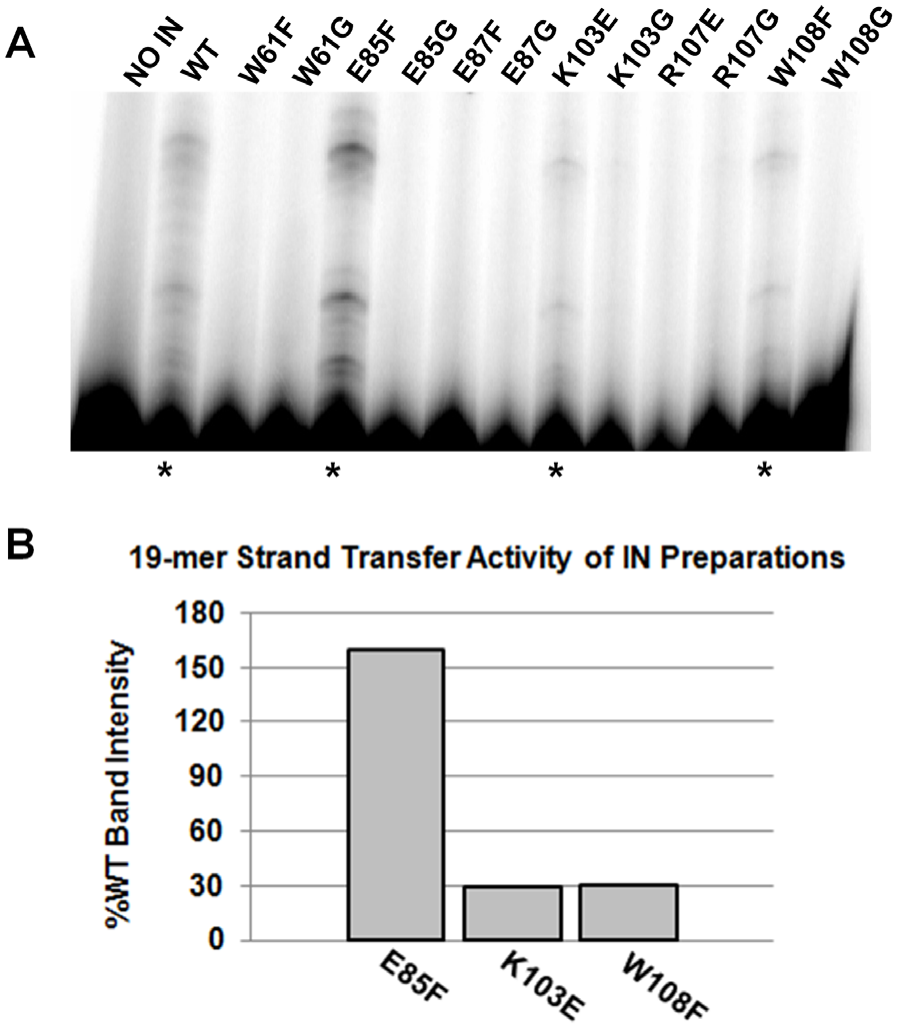
Pre-processed 19-mer DNA evokes mutant IN strand transfer activity. (A) The strand transfer activity of partly-purified mutant IN proteins with 19-mer pre-processed DNA substrate. (B) The activities of K103E and W108F are increased to about 30% of the WT levels in the presence of pre-processed 19-mer DNA substrate, while E85F is able to catalyze 160% of the WT level of integration (highlighted with an asterisk).

Since the *in vivo* strand transfer activity of IN depends directly upon the initial 3′-processing of the viral DNA substrate, we proceeded to analyze the precise catalytic point at which the interface mutants were deficient. It is plausible that, due to a minor imperfection in dimer formation leading to failed higher-order oligomerization, a mutant IN protein could be incapable of structurally progressing to a conformation conducive to proper strand transfer. In this scenario the *in vitro* strand transfer activity of this IN protein could potentially be salvaged using a pre-processed DNA substrate. To determine if any of our mutant IN proteins exhibited this phenotype, we conducted an *in vitro* enzymatic assay substituting a pre-processed 19-mer DNA substrate for the normal 21-mer (Figure 5A). Interestingly, the previously inactive K103E and W108F IN mutants displayed low-level ability (29% and 30% of WT, respectively) to integrate the 19-mer oligonucleotide, as shown in Figure 5B. This upsurge in catalytic activity suggests that the K103E and W108F IN mutants are ultimately inactive due to structural defects contributing to improper multimeric assembly. At this stage we cannot rule out the possibility that the slower 3′ OH processing activity in comparison to strand transfer activity may contribute to this differences. The E85F mutant was also proficient at integrating pre-processed DNA, at a level 1.6-fold greater than that of the WT enzyme.

### Catalytically Inactive Interface Mutants are Unable to Form Multimeric Assemblies

**Figure 6:**
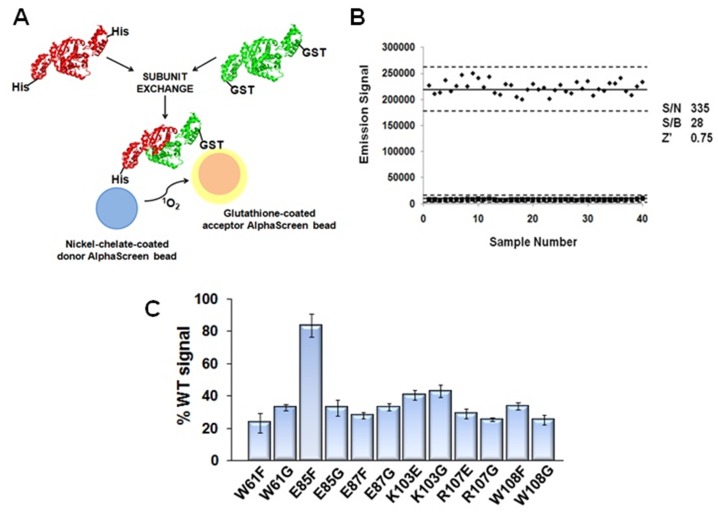
A quantitative IN subunit exchange assay specifically illustrates multimerization defect of IN dimeric interface mutants. (A) Mechanics of the subunit exchange assay for quantifying HIV-1 IN multimerization. Mutant IN was expressed with a 6x Histidine tag, while WT IN was expressed with a GST tag. Mutant IN proteins with functional oligomerization were capable of carrying out subunit exchange with WT IN, resulting in a hybrid subunit protein containing each tag. Subunit assembly allowed energy transfer between donor and acceptor beads and emission of luminescence. (B) Z-factor calculation for AlphaScreen-based multimerization assay – reproducibility of the assay deemed “excellent” with score of 0.75. Signal-to-noise and signal-to-background ratios are optimally high, at 335 and 28, respectively. Solid lines denote mean of positive control and background signal, and dotted lines represent three standard deviations from each data set. (C) Application of the multimerization assay to the measurement of mutant IN multimerization, relative to that of the WT protein. Catalytically inactive IN mutant proteins exhibited around 20–30% of WT multimerization capacity, but only the catalytically active E85F mutant protein approached WT levels of multimerization.

After corroborating the importance of the interface residues in IN catalysis, we sought to prove that the IN mutants’ lack of activity is specifically due to a block in IN oligomerization. To determine this, we used an efficient AlphaScreen®-based assay [49] that can be used to analyze total IN multimerization (shown in Figure 6A). AlphaScreen® technology relies upon chemical energy transfer between two separate beads (a donor and acceptor bead) that are each coated with different functional groups. When the donor bead is illuminated at 680 nm, it can excite an oxygen diatom to its electron singlet state, which can subsequently stimulate the acceptor bead to emit light at 570 nm. For this energy transfer to occur, the donor and acceptor beads must be within a distance of 200 nm of each other. As shown previously IN dimers readily exchange subunits in solution [53], and it follows that functional multimerization of these dimers is a prerequisite for successful subunit exchange. To test the importance of dimeric interface residues in total IN multimerization, we cloned the full-length IN protein into an N-terminally GST-tagged GEX vector, while C-terminally 6x His-tagged mutant IN proteins were used as the second necessary chemical functionality. AlphaScreen donor beads were glutathione-coated, while acceptor beads were Ni^2+^-chelate-coated. Successful subunit exchange resulted in a mixture of IN species that included an IN hybrid multimer, composed of both GST-tagged and 6xHis-tagged subunits. This hybrid was capable of binding to both donor and acceptor beads, resulting in energy transfer and acceptor bead emission. To judge the reproducibility of the assay, we calculated its Z-factor, which is a coefficient reflective of the assay’s signal dynamic range and data variation [54]. This calculation essentially compares the standard deviation to the mean of positive (maximum signal) controls and background signal controls. Here, a reaction mixture containing GST-IN and His-IN served as our positive control, while a reaction mixture containing only AlphaScreen beads was used as the background signal control. A Z-factor between 0.5 and 1.0 is generally considered representative of an “excellently” reproducible assay. The Z-factor we attained for our assay is 0.75 (Figure 6B), and signal-to-noise and signal-to-background ratios were also optimally high, with ratios of 335 and 28, respectively. Confident with the reliability of the assay, we determined the multimerization capacity of each IN protein containing substitutions at the dimerization interface. As shown in Figure 6C, the only mutant IN protein able to multimerize near WT levels was E85F, agreeing with our previous observation that this was the only mutant protein exhibiting strand transfer activity in the non-processed 21-mer *in vitro* assay. The levels of IN mutant multimerization trend similarly to the 3′-processing activity illustrated in Figure 4C. These data clearly illustrate that the substitutions we have studied at the IN dimerization interface yield defective proteins, due specifically to their impaired ability to multimerize.

### HIV-1 Virus Containing IN Substitutions at the Dimeric Interface are Deficient for Replication

**Figure 7:**
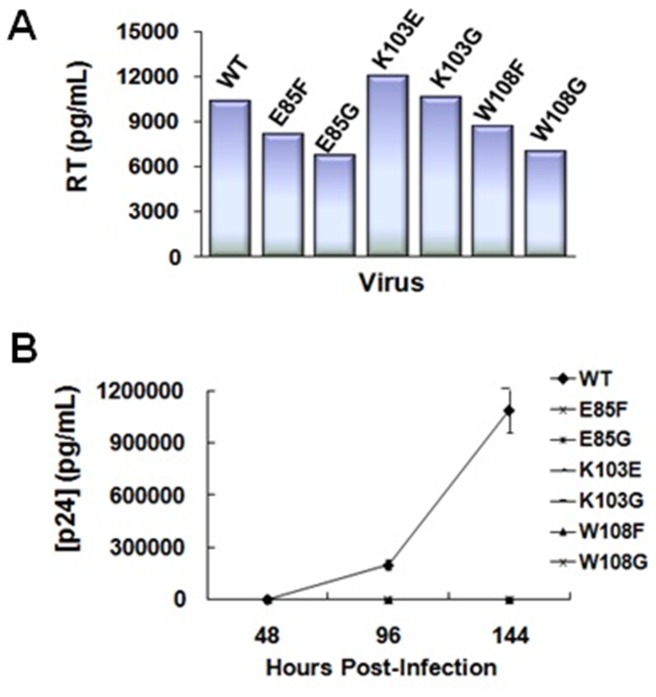
IN dimerization interface mutations inhibit HIV-1 replication after early stage. (A) Exogenous reverse transcriptase activity assay of WT and mutant NL4.3 viruses. (B) Viral breakthrough assay measuring replicative competence over a course of six days. WT virus properly reproduced to reach a p24 measurement of >1×10^6 ^pg/mL at the 144 hour time point, while each mutant virus was incompetent for viral replication.

**Figure 8:**
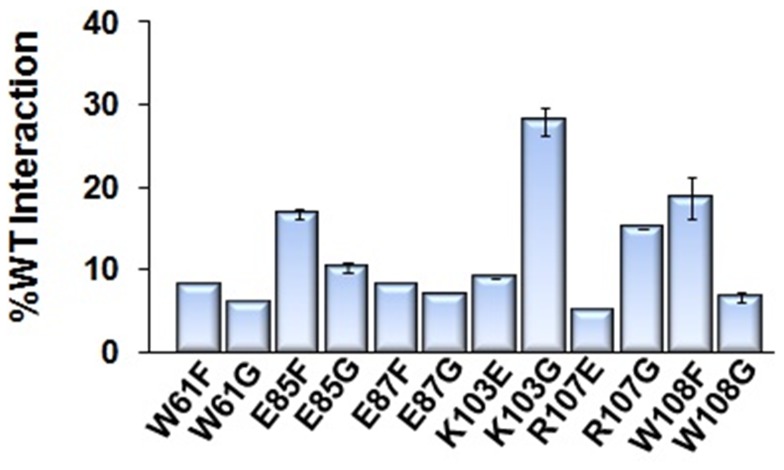
IN dimerization interface mutants exhibit reduced affinity for LEDGF/p75 *in vitro*. An AlphaScreen assay measuring mutant IN interaction with LEDGF/p75, as compared to that of the WT IN-LEDGF/p75 interaction, showed that none of the mutant IN proteins substantially interact with LEDGF/p75. The highest amount of interaction was between K103G and LEDGF/p75, but this protein still only reached about 30% of the WT interaction level.

Given that IN mutants deficient for functional multimerization are unable to catalyze strand transfer *in vitro*, we finally aimed to determine if the same IN residues are essential for viral replication. First, we produced WT and mutant HIV-1 viruses in the context of the NL4.3 infectious clone. We focused our analysis on the IN residues E85, K103, and W108, given their interesting enzymatic profile as described above. Second, since it widely known that IN mutations can have pleiotropic effects [55], we investigated whether or not IN dimeric interface mutations had any effect on exogenous HIV-1 reverse transcriptase (RT) activity. We observed that the RT activity within K103E, K103G, and W108F mutant viruses possessed near WT levels of oligonucleotide synthesis, while those of E85G and W108G exhibited a mild reduction (Figure 7A). We then conducted a viral breakthrough assay in order to compare IN mutant versus WT virus replicative capacity. We were interested to find that all viruses containing an IN substitution - including E85F - at the dimeric interface were deficient for replication in comparison to WT virus after 6 days (Figure 7B). Though the fate of E85F is not in direct agreement with our *in vitro* data, the lack of this virus’s capacity to replicate is quite plausible when considering the weakening effect that the above mutation would exert upon the structural alignment of the dimeric interface. Only strong enough to catalyze 30–40% of the WT IN level of 3′-processing and strand transfer *in vitro*, the E85F multimer may not contain the structural integrity to withstand the forces involved with viral replication as a whole, including virus-host cofactor interactions and nuclear translocation. In fact, with this possibility in mind, we screened each of the IN mutants in our panel for possible binding deficiencies with LEDGF/p75 (known to bind to an adjacent location along the IN dimeric interface) using the AlphaScreen® assay as previously described [32]. Each mutant exhibited less than one-third of the WT interaction with this cellular cofactor (Figure 8).

## Discussion

In this study we have validated a series of HIV-1 IN non-active site residues as being structurally important for IN multimerization, catalysis, and viral replication as a whole. These residues form a highly symmetric amino acid network between both IN monomers in the dimeric complex. Specifically, W61 and W108 from each monomer form a four-tiered stacking interaction capable of donating stabilization energy of ≤ −10 kcal/mol to the IN dimeric complex. Flanking this four-tiered aromatic interaction are two highly charged salt bridges composed of E85–R107 and E87–K103 (see Figure 1). Conservative and non-conservative substitutions yielded strand transfer-defective proteins, with the exception of the E85F, K103E, and W108F IN proteins. E85F exhibited near WT levels of strand transfer catalytic activity in vitro, but the K103E and W108F IN mutants showed moderate activity only in the presence of a pre-processed 19-mer substrate. We postulate that the conformations of IN residues 103 and 108 are crucial for proper multimerization leading to strand transfer catalysis. While the change from a tryptophan to a phenylalanine at position 108 is less than drastic, the positive to negative charge replacement (K to E) at position 103 is a severe modification for this charged center. It is possible that a negative charge at position 103 could repel E87 and lead to a dimeric conformation non-conducive to progression from 3′-processing to strand transfer. Other inactivating mutations from our panel appear to damage the IN multimer beyond such repair.

In order to study the effect of each substitution upon IN quaternary structure, we used a quantitative AlphaScreen®-based assay capable of monitoring IN multimerization. This is the first time that a quantitative assay has been utilized for characterization of the multimerization dynamics of HIV-1 IN mutants. Application of this assay to our full-length mutant proteins demonstrated that E85F, the only catalytically active IN protein for strand transfer, was able to multimerize near WT levels. As most of the other mutant IN proteins studied in this context were highly insoluble, we cannot specifically conclude from the AlphaScreen assay that multimerization is negatively affected. Rather, we can say that the presumably aggregating IN proteins are unable to exchange monomers in this assay.

**Figure 9:**
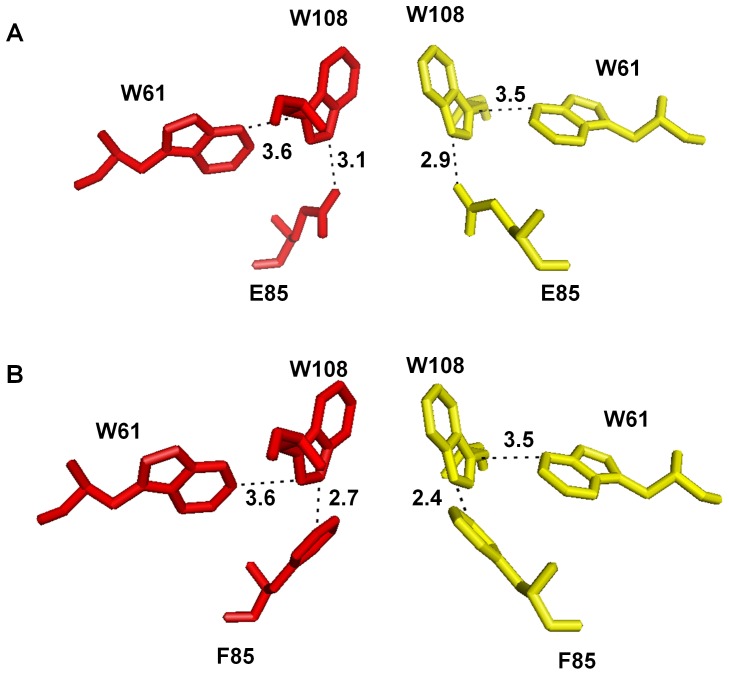
PyMOL representation of Å distance between W108 and both W61 and E85. (A) E85 comes within 2.9 Å of W108 (0.6 Å closer than W61) but also is involved in a stable salt bridge with R107. (B) The substitution of phenylalanine at position E85 brings the residue 0.5 Å closer to W108, resulting in a ∼30% shift of the residue closer to W108, as compared to the distance from W61 to W108. This distance may be conducive to stabilization of the aromatic electron orbital interactions, thus stabilizing the dimeric interface interaction.

The fact that a phenylalanine substitution of the glutamic acid at position 85 yields an active IN protein was an unexpected outcome. The substitution to a bulky, neutrally charged side-chain at this position would be expected to disrupt the vital salt bridge and generate a protein severely impaired for oligomerization, but we have observed the opposite result. Previously, a similar observation was reported in a HIV-2 IN study in which E85 was substituted with a tryptophan residue to yield an active protein [56]. Another study, substituting the same position in HIV-1 IN with alanine, produced a highly active enzyme that retained the ability to interact with LEDGF/p75 [57]. Our glycine substitution at this position, however, was clearly strand transfer-defective. The additional pi-orbital aromaticity of E85F may contribute stability to the dimeric complex through cooperation with the nearby multi-tryptophan interaction of W61 and W108. In fact, the closest distance between E85 and W108 is about 2.9 Å, while that of W61 and W108 is 3.5 Å (Figure 9A). It is plausible that F85 could join the existing aromatic interaction between IN dimers, or even seize responsibility from W61 in the scheme of the four-tiered aromatic complex. A rudimentary substitution of glutamate 85 for a phenylalanine in PyMOL yielded the observation that the side chain advances to a mere 2.4 Å from W108, or about 30% closer than W61 (Figure 9B).

After producing mutant NL4.3 HIV-1 viruses, we found that exogenous HIV-1 RT activity for all IN mutant viruses was near WT levels, signifying proper viral assembly for each virus produced. A breakthrough assay showed that all IN mutant viruses were replication-incompetent after six days in comparison to WT virus. A phenylalanine substitution at position 85, although sufficient for catalytic activity *in vitro*, results in a clear block in viral replication. It is possible that this substitution alters the conformation of an unidentified cellular cofactor binding site, or the interaction with a viral and/or cellular protein in the PIC. In fact, we observed that all IN mutations significantly reduced the proteins’ interaction with cellular binding partner LEDGF. E85F IN experienced less than 20% of the WT IN-LEDGF binding.

### Conclusions

Several efforts have aimed at disrupting the IN dimeric interface using synthetic interfacial peptides reproducing the sequences of the alpha-helices and the β-strand involved in dimerization [42], [43], [58], [59], [60]. Though moderate success has been obtained in these studies, our interests are in developing more drug-like small molecule compounds that potently inhibit IN dimerization and are also useful for clinical evaluation. To this end we have validated the use of an AlphaScreen®-based assay capable of high-throughput drug screening, and we have already begun screening our library of diverse small-molecules in order to uncover drug-like leads in this new class of IN dimerization allosteric inhibitors. Furthermore, we are undertaking rational, structure-based drug design efforts to specifically target the residues identified as essential for multimerization in this study, as well as potential nearby pockets lining the dimerization interface. Mildly-active inhibitors of HIV-1 IN dimerization have recently reported [45], and the most active of these compounds interestingly binds to the precise region characterized in this study.

We have shown that there is a very low tolerance for amino acid variation at the symmetrical dimeric interfacial region characterized in this study, and therefore drugs designed to target the amino acid network detailed here could be expected to yield a significantly reduced number of drug-resistant escape mutations compared to contemporary clinically-evaluated antiretrovirals. Importantly, we have demonstrated that a 10–15% disruption in IN multimerization is enough to reduce the enzyme’s catalytic activity by four times that amount.

## Methods

### Site-directed Mutagenesis

Site-directed mutagenesis was carried out upon the pET-15b-IN plasmid, a generous gift from Dr. Robert Craigie, Laboratory of Molecular Biology, NIDDK, NIH, Bethesda, MD, as previously described. The oligonucleotide primers were designed as follows: W61F: sense – CTAGCTGGAATATTCCTG, antisense - CAGGAATATTCCAGCTAG; W61G: sense - CTAGCTGTCCTATTCCTG, antisense - CAGGAATAGGACAGCTAG; E85F: sense – CTTCTGCGAATATATATCC, antisense - GGATATATATTCGCAGAAG; E85G: sense – CTTCTGCTCCTATATATCC, antisense - GGATATATAGGAGCAGAAG; E87F: sense – GCTGGAATTACGAATGCTTC, antisense - GAAGCATTCGTAATTCCAGC; E87G: sense – GCTGGAATTACTCCTGCTTC, antisense - GAAGCAGGAGTAATTCCAGC; K103E: sense – GGCCATCTTCCTGCTAATTCTAAGAGG, antisense - CCTCTTAGAATTAGCAGGAAGATGGCC; K103G: sense – GGCCATCTTCCTGCTAATCCTAAGAGG, antisense - CCTCTTAGGATTAGCAGGAAGATGGCC; R107E: sense – ACTGGCCATTCTCCTGC, antisense - GCAGGAGAATGGCCAGT; R107G: sense – ACTGGCCATCCTCCTGC, antisense - GCAGGAGGATGGCCAGT; W108F: sense – GTTTTTACTGGGAATCTTCCTGC, antisense - GCAGGAAGATTCCCAGTAAAAAC; W108G: sense – GTTTTTACTGGTCCTCTTCCTGC, antisense - GCAGGAAGAGGACCAGTAAAAAC. Nucleotide mutations were confirmed by DNA sequencing at the USC/Norris Comprehensive Cancer Center Microchemical Core Facility (University of Southern California).

### Expression of HIV-1 IN lysates (Isolation and Processing of Inclusion Bodies)

The HIV-1 IN plasmid was transformed with heat shock into BL21 (DE3) pLYSs expression strain *Escherichia coli* (Invitrogen) and allowed to grow to optical density 0.75 (595 nm) before induction with 1 mM IPTG. Culture was then allowed to grow for an additional three hours at 37°C and 250 rpm shaking. Cells were then centrifuged for 20 minutes at 3000 rpm. Pelleted cells were resuspended in lysis buffer (20 mM HEPES, pH 7.5; 5 mM imidazole; 100 mM NaCl) and passed twice through a French® Pressure Cell Press (Thermo Spectronic). Lysate was centrifuged at 31,000 *g*, and pellet was resuspended in a solubilization buffer (20 mM HEPES, pH 7.5; 5 mM imidazole; 1 M NaCl, 1 mM CHAPS). Lysate was then dialyzed in Spectra/Por molecular porous membrane tubing, MWCO 12-14,000 (Spectrum Laboratories, Inc.), suspended in a buffer containing 20 mM HEPES, pH 7.5; 500 mM NaCl; 40% glycerol; 0.2 mM EDTA, and 1 mM dithiothreitol. For protein concentration determination, aliquots of protein were via PAGE, along with a BSA standard curve of 10 mg/mL, 5 mg/mL, 2.5 mg/mL, 1.25 mg/mL, and 0.6 mg/mL. The gel was photographed with a Typhoon 8610 Variable Mode Imager (Amersham Biosciences), and band intensity was measured by densitometric analysis using ImageJ software. The standard curve equation obtained from the BSA band intensities was used to calculate the protein concentrations of each IN band in the processed cellular extracts.

### Oligonucleotide Subtrates and IN Enzymatic Assays

As previously described [48]. MnCl_2_ was used as the metal cofactor in this study.

### AlphaScreen® Multimerization Assay

IN extracts were dissolved in reaction buffer (150 mM NaCl, 25 mM Tris-HCl pH 7.3, 1 mM MgCl_2_, 0.1% Tween 20, 0.1% BSA). A sample volume of 25 µL was used in the 384 well format. GST-tagged WT IN was incubated in equimolar concentration with 6xHis-tagged mutant IN at 4°C overnight. The next morning, glutathione-coated donor beads and Ni^2+^-coated acceptor beads (PerkinElmer) were added at a final concentration of 1 mg/mL. The plate was incubated at 30°C for one hour and then scanned on an EnVision™ plate reader (PerkinElmer).

#### Viral strain

The HIV-1 molecular clone pNL4.3 was obtained through the AIDS Research and Reference Reagent Program, Division of AIDS, NIAID, NIH contributed by Dr. Malcolm Martin (Bethesda, MD). To generate IN mutant viruses, site-directed mutagenesis was performed using the Kirsch and Joly method [61]. The presence of the expected mutations was confirmed by DNA sequencing of the entire IN coding region. Virus productions were performed as described previously [62]. Briefly, 6 million 293T cells were transfected with WT or mutagenized NL4.3 plasmid using branched PEI. Virus was harvested and filtered 48 hours later, and p24 antigen measurements were taken for quantification of viral titer.

### HIV-1 RT Activity Assay

Viral RT nucleotide incorporation was quantified per kit instructions provided by manufacturer: HS-Lenti Kit-RT assay (Cavidi, Sweden).

### HIV-1 Breakthrough Infection Assay

Twenty-thousand HeLa P4 cells in 0.5 mL RPMI medium (12% FCS) were seeded per well in 6-well plates. Virus corresponding to 50 pg p24/mL (Alliance HIV-1 P24 antigen ELISA Kit, Perkin Elmer Life Sciences, Milano Italy) was added to the cells and incubated overnight at 37°C. Aliquots of cell-free supernatants were harvested for determination of viral p24 levels at two days, four days, and six days post-infection.

### AlphaScreen® Assay for Measuring IN-LEDGF/p75 Interaction

Assay was performed as previously described [32], with mutant IN proteins substituted for the WT version of the protein.

#### Accession Numbers

The Genbank (http://www.ncbi.nlm.nih.gov.Genbank) accession numbers for the proteins discussed in this paper are LEDGF/p75 (NM_033222) and HIV-1 NL4-3 IN (U26942).

## References

[1] Mouscadet JF, Delelis O, Marcelin AG, Tchertanov L (2010) Resistance to HIV-1 integrase inhibitors: A structural perspective. Drug Resist Updat 13: 139–150. 20570551 10.1016/j.drup.2010.05.001

[2] Mouscadet JF, Tchertanov L (2009) Raltegravir: molecular basis of its mechanism of action. Eur J Med Res 14 Suppl 35–16. 19959411 10.1186/2047-783X-14-S3-5 PMC3516820

[3] Ramkumar K, Serrao E, Odde S, Neamati N (2010) HIV-1 integrase inhibitors: 2007–2008 update. Med Res Rev 30: 890–954. 20135632 10.1002/med.20194

[4] Serrao E, Odde S, Ramkumar K, Neamati N (2009) Raltegravir, elvitegravir, and metoogravir: the birth of “me-too” HIV-1 integrase inhibitors. Retrovirology 6: 25. 19265512 10.1186/1742-4690-6-25 PMC2660292

[5] Coiras M, Lopez-Huertas MR, Sanchez del Cojo M, Mateos E, Alcami J (2010) Dual role of host cell factors in HIV-1 replication: restriction and enhancement of the viral cycle. AIDS Rev 12: 103–112. 20571604

[6] Kinoshita S, Chen BK, Kaneshima H, Nolan GP (1998) Host control of HIV-1 parasitism in T cells by the nuclear factor of activated T cells. Cell 95: 595–604. 9845362 10.1016/s0092-8674(00)81630-x

[7] Tardif MR, Tremblay MJ (2005) Tetraspanin CD81 provides a costimulatory signal resulting in increased human immunodeficiency virus type 1 gene expression in primary CD4+ T lymphocytes through NF-kappaB, NFAT, and AP-1 transduction pathways. J Virol 79: 4316–4328. 15767432 10.1128/JVI.79.7.4316-4328.2005 PMC1061526

[8] Chun TW, Justement JS, Lempicki RA, Yang J, Dennis G Jr, et al (2003) Gene expression and viral prodution in latently infected, resting CD4+ T cells in viremic versus aviremic HIV-infected individuals. Proc Natl Acad Sci U S A 100: 1908–1913. 12552096 10.1073/pnas.0437640100 PMC149932

[9] Chun TW, Nickle DC, Justement JS, Large D, Semerjian A, et al (2005) HIV-infected individuals receiving effective antiviral therapy for extended periods of time continually replenish their viral reservoir. J Clin Invest 115: 3250–3255. 16276421 10.1172/JCI26197 PMC1265878

[10] Furtado MR, Callaway DS, Phair JP, Kunstman KJ, Stanton JL, et al (1999) Persistence of HIV-1 transcription in peripheral-blood mononuclear cells in patients receiving potent antiretroviral therapy. N Engl J Med 340: 1614–1622. 10341273 10.1056/NEJM199905273402102

[11] Yerly S, Perneger TV, Vora S, Hirschel B, Perrin L (2000) Decay of cell-associated HIV-1 DNA correlates with residual replication in patients treated during acute HIV-1 infection. AIDS 14: 2805–2812. 11153661 10.1097/00002030-200012220-00001

[12] Engelman A, Bushman FD, Craigie R (1993) Identification of discrete functional domains of HIV-1 integrase and their organization within an active multimeric complex. EMBO J 12: 3269–3275. 8344264 10.1002/j.1460-2075.1993.tb05996.x PMC413594

[13] Fletcher TM 3rd, Soares MA, McPhearson S, Hui H, Wiskerchen M, et al (1997) Complementation of integrase function in HIV-1 virions. EMBO J 16: 5123–5138. 9305653 10.1093/emboj/16.16.5123 PMC1170146

[14] van den Ent FM, Vos A, Plasterk RH (1999) Dissecting the role of the N-terminal domain of human immunodeficiency virus integrase by trans-complementation analysis. J Virol 73: 3176–3183. 10074170 10.1128/jvi.73.4.3176-3183.1999 PMC104080

[15] Chen JC, Krucinski J, Miercke LJ, Finer-Moore JS, Tang AH, et al (2000) Crystal structure of the HIV-1 integrase catalytic core and C-terminal domains: a model for viral DNA binding. Proc Natl Acad Sci U S A 97: 8233–8238. 10890912 10.1073/pnas.150220297 PMC26930

[16] Sluis-Cremer N, Tachedjian G (2002) Modulation of the oligomeric structures of HIV-1 retroviral enzymes by synthetic peptides and small molecules. Eur J Biochem 269: 5103–5111. 12392542 10.1046/j.1432-1033.2002.03216.x

[17] Hamamoto S, Nishitsuji H, Amagasa T, Kannagi M, Masuda T (2006) Identification of a novel human immunodeficiency virus type 1 integrase interactor, Gemin2, that facilitates efficient viral cDNA synthesis in vivo. J Virol 80: 5670–5677. 16731905 10.1128/JVI.02471-05 PMC1472599

[18] Jenkins TM, Engelman A, Ghirlando R, Craigie R (1996) A soluble active mutant of HIV-1 integrase: involvement of both the core and carboxyl-terminal domains in multimerization. J Biol Chem 271: 7712–7718. 8631811 10.1074/jbc.271.13.7712

[19] Luo K, Wang T, Liu B, Tian C, Xiao Z, et al (2007) Cytidine deaminases APOBEC3G and APOBEC3F interact with human immunodeficiency virus type 1 integrase and inhibit proviral DNA formation. J Virol 81: 7238–7248. 17428847 10.1128/JVI.02584-06 PMC1933265

[20] Farnet CM, Haseltine WA (1991) Determination of viral proteins present in the human immunodeficiency virus type 1 preintegration complex. J Virol 65: 1910–1915. 2002549 10.1128/jvi.65.4.1910-1915.1991 PMC240011

[21] Wu X, Liu H, Xiao H, Conway JA, Hehl E, et al (1999) Human immunodeficiency virus type 1 integrase protein promotes reverse transcription through specific interactions with the nucleoprotein reverse transcription complex. J Virol 73: 2126–2135. 9971795 10.1128/jvi.73.3.2126-2135.1999 PMC104457

[22] Pauza CD (1990) Two bases are deleted from the termini of HIV-1 linear DNA during integrative recombination. Virology 179: 886–889. 2238479 10.1016/0042-6822(90)90161-j

[23] De Rijck J, Vandekerckhove L, Christ F, Debyser Z (2007) Lentiviral nuclear import: a complex interplay between virus and host. Bioessays 29: 441–451. 17450594 10.1002/bies.20561

[24] Llano M, Vanegas M, Fregoso O, Saenz D, Chung S, et al (2004) LEDGF/p75 determines cellular trafficking of diverse lentiviral but not murine oncoretroviral integrase proteins and is a component of functional lentiviral preintegration complexes. J Virol 78: 9524–9537. 15308744 10.1128/JVI.78.17.9524-9537.2004 PMC506940

[25] Maertens G, Cherepanov P, Pluymers W, Busschots K, De Clercq E, et al (2003) LEDGF/p75 is essential for nuclear and chromosomal targeting of HIV-1 integrase in human cells. J Biol Chem 278: 33528–33539. 12796494 10.1074/jbc.M303594200

[26] Schroder AR, Shinn P, Chen H, Berry C, Ecker JR, et al (2002) HIV-1 integration in the human genome favors active genes and local hotspots. Cell 110: 521–529. 12202041 10.1016/s0092-8674(02)00864-4

[27] Gijsbers R, Ronen K, Vets S, Malani N, De Rijck J, et al (2010) LEDGF hybrids efficiently retarget lentiviral integration into heterochromatin. Mol Ther 18: 552–560. 20195265 10.1038/mt.2010.36 PMC2839429

[28] Holman AG, Coffin JM (2005) Symmetrical base preferences surrounding HIV-1, avian sarcoma/leukosis virus, and murine leukemia virus integration sites. Proc Natl Acad Sci U S A 102: 6103–6107. 15802467 10.1073/pnas.0501646102 PMC1087937

[29] Wu X, Li Y, Crise B, Burgess SM, Munroe DJ (2005) Weak palindromic consensus sequences are a common feature found at the integration target sites of many retroviruses. J Virol 79: 5211–5214. 15795304 10.1128/JVI.79.8.5211-5214.2005 PMC1069554

[30] Engelman A, Craigie R (1992) Identification of conserved amino acid residues critical for human immunodeficiency virus type 1 integrase function in vitro. J Virol 66: 6361–6369. 1404595 10.1128/jvi.66.11.6361-6369.1992 PMC240128

[31] Kulkosky J, Jones KS, Katz RA, Mack JP, Skalka AM (1992) Residues critical for retroviral integrative recombination in a region that is highly conserved among retroviral/retrotransposon integrases and bacterial insertion sequence transposases. Mol Cell Biol 12: 2331–2338. 1314954 10.1128/mcb.12.5.2331-2338.1992 PMC364405

[32] Christ F, Voet A, Marchand A, Nicolet S, Desimmie BA, et al (2010) Rational design of small-molecule inhibitors of the LEDGF/p75-integrase interaction and HIV replication. Nat Chem Biol 6: 442–448. 20473303 10.1038/nchembio.370

[33] De Luca L, Barreca ML, Ferro S, Christ F, Iraci N, et al (2009) Pharmacophore-based discovery of small-molecule inhibitors of protein-protein interactions between HIV-1 integrase and cellular cofactor LEDGF/p75. ChemMedChem 4: 1311–1316. 19565598 10.1002/cmdc.200900070

[34] Kessl JJ, Eidahl JO, Shkriabai N, Zhao Z, McKee CJ, et al (2009) An allosteric mechanism for inhibiting HIV-1 integrase with a small molecule. Mol Pharmacol 76: 824–832. 19638533 10.1124/mol.109.058883 PMC2769043

[35] Fitzkee NC, Masse JE, Shen Y, Davies DR, Bax A (2010) Solution conformation and dynamics of the HIV-1 integrase core domain. J Biol Chem 285: 18072–18084. 20363759 10.1074/jbc.M110.113407 PMC2878568

[36] Fitzkee NC, Torchia DA, Bax A (2011) Measuring rapid hydrogen exchange in the homodimeric 36 kDa HIV-1 integrase catalytic core domain. Protein Sci 20: 500–512. 21213249 10.1002/pro.582 PMC3064830

[37] Sippel M, Sotriffer CA (2010) Molecular dynamics simulations of the HIV-1 integrase dimerization interface: guidelines for the design of a novel class of integrase inhibitors. J Chem Inf Model 50: 604–614. 20230013 10.1021/ci900403s

[38] Armon-Omer A, Levin A, Hayouka Z, Butz K, Hoppe-Seyler F, et al (2008) Correlation between shiftide activity and HIV-1 integrase inhibition by a peptide selected from a combinatorial library. J Mol Biol 376: 971–982. 18201721 10.1016/j.jmb.2007.11.095

[39] Hayouka Z, Rosenbluh J, Levin A, Loya S, Lebendiker M, et al (2007) Inhibiting HIV-1 integrase by shifting its oligomerization equilibrium. Proc Natl Acad Sci U S A 104: 8316–8321. 17488811 10.1073/pnas.0700781104 PMC1895947

[40] Kong R, Wang C, Ma X, Liu J, Chen W (2005) Peptides design based on the interfacial helix of integrase dimer. Conf Proc IEEE Eng Med Biol Soc 5: 4743–4746. 17281301 10.1109/IEMBS.2005.1615531

[41] Krajewski K, Long YQ, Marchand C, Pommier Y, Roller PP (2003) Design and synthesis of dimeric HIV-1 integrase inhibitory peptides. Bioorg Med Chem Lett 13: 3203–3205. 12951093 10.1016/s0960-894x(03)00679-6

[42] Maroun RG, Gayet S, Benleulmi MS, Porumb H, Zargarian L, et al (2001) Peptide inhibitors of HIV-1 integrase dissociate the enzyme oligomers. Biochemistry 40: 13840–13848. 11705373 10.1021/bi011328n

[43] Zhao L, O’Reilly MK, Shultz MD, Chmielewski J (2003) Interfacial peptide inhibitors of HIV-1 integrase activity and dimerization. Bioorg Med Chem Lett 13: 1175–1177. 12643937 10.1016/s0960-894x(03)00040-4

[44] Abdel-Malek S, Bastien JW, Mahler WF, Jia Q, Reinecke MG, et al (1996) Drug leads from the Kallawaya herbalists of Bolivia. 1. Background, rationale, protocol and anti-HIV activity. J Ethnopharmacol 50: 157–166. 8691850 10.1016/0378-8741(96)01380-3

[45] Tintori C, Demeulemeester J, Franchi L, Massa S, Debyser Z, et al.. (2012) Discovery of small molecule HIV-1 integrase dimerization inhibitors. Bioorg Med Chem Lett. 10.1016/j.bmcl.2012.03.064 22483582

[46] Vassilev LT, Vu BT, Graves B, Carvajal D, Podlaski F, et al (2004) In vivo activation of the p53 pathway by small-molecule antagonists of MDM2. Science 303: 844–848. 14704432 10.1126/science.1092472

[47] Al-Mawsawi LQ, Fikkert V, Dayam R, Witvrouw M, Burke TR Jr, et al (2006) Discovery of a small-molecule HIV-1 integrase inhibitor-binding site. Proc Natl Acad Sci U S A 103: 10080–10085. 16785440 10.1073/pnas.0511254103 PMC1502509

[48] Al-Mawsawi LQ, Hombrouck A, Dayam R, Debyser Z, Neamati N (2008) Four-tiered pi interaction at the dimeric interface of HIV-1 integrase critical for DNA integration and viral infectivity. Virology 377: 355–363. 18514248 10.1016/j.virol.2008.04.030

[49] Demeulemeester J, Tintori C, Botta M, Debyser Z, Christ F (2012) Development of an AlphaScreen-Based HIV-1 Integrase Dimerization Assay for Discovery of Novel Allosteric Inhibitors. J Biomol Screen. 10.1177/1087057111436343 22337657

[50] Pandey KK, Bera S, Grandgenett DP (2011) The HIV-1 integrase monomer induces a specific interaction with LTR DNA for concerted integration. Biochemistry 50: 9788–9796. 21992419 10.1021/bi201247f PMC3212032

[51] Lee SP, Xiao J, Knutson JR, Lewis MS, Han MK (1997) Zn2+ promotes the self-association of human immunodeficiency virus type-1 integrase in vitro. Biochemistry 36: 173–180. 8993331 10.1021/bi961849o

[52] Zheng R, Jenkins TM, Craigie R (1996) Zinc folds the N-terminal domain of HIV-1 integrase, promotes multimerization, and enhances catalytic activity. Proc Natl Acad Sci U S A 93: 13659–13664. 8942990 10.1073/pnas.93.24.13659 PMC19383

[53] Cai M, Zheng R, Caffrey M, Craigie R, Clore GM, et al (1997) Solution structure of the N-terminal zinc binding domain of HIV-1 integrase. Nat Struct Biol 4: 567–577. 9228950 10.1038/nsb0797-567

[54] Zhang JH, Chung TD, Oldenburg KR (1999) A Simple Statistical Parameter for Use in Evaluation and Validation of High Throughput Screening Assays. J Biomol Screen 4: 67–73. 10838414 10.1177/108705719900400206

[55] Engelman A, Englund G, Orenstein JM, Martin MA, Craigie R (1995) Multiple effects of mutations in human immunodeficiency virus type 1 integrase on viral replication. J Virol 69: 2729–2736. 7535863 10.1128/jvi.69.5.2729-2736.1995 PMC188965

[56] van den Ent FM, Vos A, Plasterk RH (1998) Mutational scan of the human immunodeficiency virus type 2 integrase protein. J Virol 72: 3916–3924. 9557677 10.1128/jvi.72.5.3916-3924.1998 PMC109617

[57] Busschots K, Voet A, De Maeyer M, Rain JC, Emiliani S, et al (2007) Identification of the LEDGF/p75 binding site in HIV-1 integrase. J Mol Biol 365: 1480–1492. 17137594 10.1016/j.jmb.2006.10.094

[58] Krajewski K, Marchand C, Long YQ, Pommier Y, Roller PP (2004) Synthesis and HIV-1 integrase inhibitory activity of dimeric and tetrameric analogs of indolicidin. Bioorg Med Chem Lett 14: 5595–5598. 15482931 10.1016/j.bmcl.2004.08.061

[59] Li HY, Zawahir Z, Song LD, Long YQ, Neamati N (2006) Sequence-based design and discovery of peptide inhibitors of HIV-1 integrase: insight into the binding mode of the enzyme. J Med Chem 49: 4477–4486. 16854053 10.1021/jm060307u

[60] Robinson WE Jr, McDougall B, Tran D, Selsted ME (1998) Anti-HIV-1 activity of indolicidin, an antimicrobial peptide from neutrophils. J Leukoc Biol 63: 94–100. 9469478 10.1002/jlb.63.1.94

[61] Kirsch RD, Joly E (1998) An improved PCR-mutagenesis strategy for two-site mutagenesis or sequence swapping between related genes. Nucleic Acids Res 26: 1848–1850. 9512562 10.1093/nar/26.7.1848 PMC147442

[62] De Rijck J, Vandekerckhove L, Gijsbers R, Hombrouck A, Hendrix J, et al (2006) Overexpression of the lens epithelium-derived growth factor/p75 integrase binding domain inhibits human immunodeficiency virus replication. J Virol 80: 11498–11509. 16987986 10.1128/JVI.00801-06 PMC1642583

